# Why Do We Pursue Ed.D.?: A Qualitative Analysis on the Motivation of Chinese Candidates

**DOI:** 10.3389/fpsyg.2022.897379

**Published:** 2022-07-06

**Authors:** Wenting Gong, Weihua Wang, Chuang Xu

**Affiliations:** ^1^School of Educational Science, Hunan Normal University, Changsha, China; ^2^School of International Studies, Hunan Institute of Technology, Hengyang, China; ^3^Office of Teaching Quality Supervision and Assessment, Hunan Institute of Technology, Hengyang, China

**Keywords:** Ed.D. program, motivation, Chinese educational practitioner, grounded theory, motivational model

## Abstract

This study aims to explore what motivates Chinese mid-career educational practitioners to pursue Ed. D. A university in South China was selected as a case university, and 18 Ed.D. candidates were recruited to participate in semi-structured interviews. Grounded theory analysis was conducted on the transcripts of the interviewees' information. The findings uncovered four motivational patterns (pre-intrinsic, pre-extrinsic, post-intrinsic, and post-extrinsic) of Ed.D. candidates in China that mutually influence and reinforce one another. A theoretical model was thus constructed in which extrinsic factors moderate intrinsic factors, prepositional factors stimulate post-positional factors, with pre-intrinsic factors act as incentives, pre-extrinsic factors act as preconditions, and post-intrinsic factors and post-extrinsic factors act as internal and external reinforcers, respectively. This study broadens critical understanding of Ed.D. students' motivation and offers several implications that should be of interest to Chinese Ed.D. candidates, educational researchers, universities, and government officials.

## Introduction

Globally, professional doctorate programs have adapted to new workplace demands and become increasingly popular. Although once criticized as indistinguishable from Ph.D. degrees, professional doctorates have progressively established a tripartite relationship among the profession, academia, and students (Taylor and Maxwell, 2004) by combining discipline-based and practice-site knowledge (Butcher and Sieminski, 2006). The Doctorate in Education (Ed.D.) was one of the first professional doctorate programs to be developed and had various types across the world (Wildy et al., 2015). It is designed as acomplement to traditional Ph.D. in Education and addresses the in-service career needs of research professionals (Butcher and Sieminski, 2006).

Candidates for professional doctorate present different and complex motivations both across and within program types (Scott et al., 2004; Wellington and Sikes, 2006). Intrinsic and extrinsic motivation types are broadly discussed. Intrinsic motivation, like personal fulfillment and intellectual challenge, is considered the dominant factor in the stimulation of adult students continuing postgraduate education (Templeton, 2016). In comparison, accelerated promotion and career development are thought to be essential motives for the learner with extrinsic motivations (Scott et al., 2004). The dichotomy of motivation types emerging from professional doctorate candidates seems to be reasonable, but it fails to depict the complex and messy nature of human motivation. Furthermore, the transition of extrinsic and intrinsic motivations has been inferred since the external social context somehow nurtures intrinsic motivation and promotes internalization for students (Deci et al., 1991). Ed.D., as one of the most popular professional doctorates in the world, looks at the variability across professional doctorates (Zambo et al., 2014). The variability lies in Ed.D. candidates' experiences, and values differ for those students in conventional doctoral programs (Taylor, 2007). Hence, there is no doubt that Ed.D. candidates' motivation differs from those who pursue Ph.D. as well as the other professional doctorates. Although recent studies have examined how Ed.D. candidates are motivated, the mechanism of internal factors interacting with external ones remained largely unexplored. In addition, though there has been a growing body of research in China focused on Ed.D. programs, few studies have focused on students' motivations of pursuing Ed.D. and fewer have been published in English. To address this gap, this study focused on exploring the motivations of Ed.D. candidates in the Chinese context with the help of grounded theory.

Ed.D. programs were first awarded in China in 2010. In 2009, China's National Office for Academic Degrees issued the Plan for Professional Degree Establishment of Doctorate in Education. The plan stated that the Ed.D. was mainly designed for educational practitioners at all levels, such as school teachers, principals, teacher educators, student counselors, administrators, and policymakers. It was expected that most individuals pursuing this type of degree would be outstanding members in their respective educational fields or at least hold a middle management position. In 2010, 15 prestigious universities, such as Perking University and Tsinghua University, officially began to recruit Ed.D. students with the permission of the Chinese government. Ed.D. programs have developed rapidly in China over the last decade, with 12 more universities receiving permission to establish this degree program in 2018. In 2020, in line with China's goal of building world-class universities within decades and promoting social development, professional doctoral programs, including Ed.D, have been strategically and strongly encouraged by the Chinese government. Thus, Ed.D programs ushered in its prevalence and witnessed a double increase of candidates in 2021. China's Ed.D. programs are similar to those offered in the UK, in that they serve as a means of enhancing in-service qualifications (Butcher and Sieminski, 2006; Taylor, 2007). However, they differ from programs located in Australia, where the Ed.D. is essentially a continuation of a master's degree in educational management (Wildy et al., 2015). China's Ed.D. programs are only for mid-career educational practitioners with more than 5 years' work experience. This study aims to clarify factors motivating Chinese adult learners with full-time employment to enroll in Ed.D. programs.

## Literature Review

Harvard created the world's first Ed.D. program in 1921 as a “pre-service” training program (Andersen, 1983; Kot and Hendel, 2012). The introduction of Ed.D. programs in Australia and the UK is relatively recent in comparison, which was in 1991 (Maxwell and Shanahan, 2001; Taylor and Maxwell, 2004) and 1992 (Butcher and Sieminski, 2006), as an “in-service” program that targeted professional development (Maxwell and Shanahan, 1997; Kot and Hendel, 2012). Research on Ed.D. programs in those countries have been conducted for decades. Comparatively, the development of Ed.D. programs in Asian countries, such as China, has received little attention.

Although scholars have debated the nature of Ed.D. since its introduction at Harvard (Maxwell and Shanahan, 1997), the Ed.D. is now taking the lead over other professional programs both in terms of enrollment and graduation rates (Kot and Hendel, 2012) and has gradually established its concepts and priorities. Maxwell (2003) stated that the creation of an Ed.D. program is structurally different and has a solid professional flavor (Maxwell, 2003). Maxwell and Shanahan (1997) noted that Ed.D. programs are concerned with the production of knowledge in professionals. Similarly, when synthesizing the critical features of the Ed.D., Taylor and Maxwell (2004) emphasized that the learning occurs in the professional workplace. Boud and Lee (2009) prompted academic interests should coexist with workplace concerns. In addition, Lindsay et al. (2018) noted that the Ed.D. is a type of teacher education that emphasizes the need for knowledge, practical experience, and research literacy. In conclusion, “profession” and “workplace” are core concepts in Ed.D. programs, which implies a distinct population from the candidates for Ph.D.

Concerning the status of Ed.D., some researchers have indicated that a Ph.D. has greater prestige (Perry, 2012b), while others believe that an Ed.D. is equal to but different from a Ph.D. (Maxwell and Shanahan, 1997; Wildy et al., 2015). As a scholarly practitioner degree (Perry, 2015), Ed.D. emphasizes professional training and continuing professional development (Taylor, 2007). The aim of developing the Ed.D was to put professional practice and critical reflection at the center of the experience (Lindsay et al., 2018), or as Wildy et al. (2015) described it, to develop different skills for a “new kind of research supervision.” Because Ed.D. programs have professional affiliations and connections to the workplace (Maxwell and Shanahan, 2001), Ed.D. students generally work full time, thrive in their current roles, and want to remain in the field (Zambo et al., 2014). As Hall (Hall, 2013, p. 162) suggested, the students who join an Ed.D. program are “mid-career professional educators” and “competent learners.” For instance, Australia established the Ed.D. as an alternative and more accessible avenue by which experienced educators could engage in an advanced study (Kot and Hendel, 2012). Previous professional experience was thus a requirement for acceptance into most Ed.D. programs (Maxwell and Shanahan, 1997; Neumann, 2005). Both part-time and full-time studies were permitted to strengthen connections with the workplace. Some universities require Ed.D. applicants hold a master's degree in education or a related discipline (Lindsay et al., 2018).

As Smith (2000) explained, the popularity of the Ed.D. was because of its flexibility for part-time students and meeting demands from an increasingly credentialed population. Besides, Ed.D. programs met the professional needs of these students and blended practical wisdom into their theories and research practices (Amrein-Beardsley et al., 2012). Ed.D. programs also provide students opportunities to collaborate with others who had the same professional background and work toward the common good (Perry, 2015). Some scholars in the US initiated the Carnegie Project on the Education Doctorate (CPED) in 2007 with 86 memberships in the U.S., Canada, and New Zealand. CPED members worked to reach a consensus about the goals of Ed.D. programs. They determined that the primary objectives should be preparing educators for the application of appropriate practices, the generation of new knowledge, and stewardship of the profession (Perry, 2015). In summary, instead of training research practitioners, as a Ph.D. does, an Ed.D. aims to train practitioner researchers by requiring them to master professional knowledge and methods and creatively solve critical problems in educational practice.

Motivation is not a new topic in higher education. It concerns the direction and magnitude of learners' behavior, that is, the choice of a particular action, the persistence with it, and the effort expended on it (Estupinan Boboy, 2014). Debates concerning learner motivation, in general, are complex (Breen and Lindsay, 1999). However, several motivational frameworks were proposed to clarify the complexity. In the socio-cultural model, motivation is characterized by two orientations, intrinsic and extrinsic (Gardner et al., 1985). Intrinsic motivation is from one's innate desire and curiosity about the activity, while extrinsic motivation is governed by the goals, values, interests of others, and external, tangible rewards (Covington and Müeller, 2001). The integrative/instrumental dichotomy is also broadly discussed (Dörnyei, 1994; Estupinan Boboy, 2014). The former is associated with a positive disposition and the desire to interact with and even become similar to valued members of that community, and the latter is related to the potential gains, such as getting a better job or a higher salary. Prior studies have investigated various motivational factors in doctoral students, as Cardona (2013) reported that intrinsic, extrinsic, and autonomous motivations were important in the course of degree completion. Besides, students' personal and academic needs, the academic environment, and career and academic support would influence their motivation to complete the doctoral degree (Cardona, 2013).

Ed.D. is attractive to those individuals who view their personal growth and academic ambition as fully integrated with their professional development and commit to furthering the cause of their profession (Bourner et al., 2001, p. 81). For other candidates, the decision to pursue an Ed.D. lies in the desire to develop academic skills and gain new academic insights (Butcher and Sieminski, 2006). Scott et al. (2004) established three models of motivation that characterize professional doctoral students and form an extrinsic-intrinsic continuum dependent on job experience: extrinsic-professional initiation, extrinsic-professional continuation, and intrinsic personal/professional affirmation. Several empirical research have also proposed a diverse range of motivations of Ed.D. candidates. After examining 29 students enrolled in an Ed.D. program at the University of Sheffield, Wellington and Sikes (2006) identified the following reasons that participants decided to pursue this type of degree: to keep their job as secure as possible, to compensate for professional frustrations, to seek out knowledge and intellectual challenge, and to confirm their positive sense of identity. After investigating 14 participating CPED institutions, Zambo et al.(2014) verified that students pursue Ed.D. for professional, career-related goals (external motivation), personal goals (internal motivation), and because of the degree itself. Wildy et al. (2015) elaborated on the questions about why students enroll in Ed.D. programs from the perspective of university staff in Australia, China, and Iceland. In the Australian context, the researchers found extraordinarily high completion rates, the support provided for participants, and that substantiate professional practice played an essential role in the decision-making process. Lower entrance examination scores played a notable role in the Chinese context, and a robust culture of lifelong learning influenced students' motivations in the Icelandic context. Lindsay et al. (2018) interviewed 25 students who had completed or were currently pursuing their Ed.D. and performed a thematic analysis to illustrate the participants' perception of their development needs. Their research revealed the following motivational themes, developing research and study skills, blending theory and practice, building supportive relationships, reflecting on theory and practice, building your resilience, developing your identity, engaging with new opportunities, disseminating your research, and making a difference.

As the discussion demonstrates, researchers have spent a great deal of effort examining the emergence and development of the Ed.D. and the differences between an Ed.D. and a Ph.D.; however, the factors that motivate individuals to pursue the former doctorate instead of the latter deserve further attention. China's unique training environment and program structures make the topic more necessary to explore. In addition, most of the empirical studies that focus on the motivation of Ed.D. students only present the results of investigations and interviews, and the internal mechanism and structure need to be further analyzed and constructed. This study uses the qualitative research method of grounded theory to study the factors that motivate Chinese domestic Ed.D. candidates with the new exploration of research methods and theoretical frameworks.

## Methodology

### Approach

This study intends to explore the driving factors of why Chinese mid-career educational practitioners pursue an Ed.D. degree and how they perceive that motivation. Because few studies have examined the cause of Ed.D. applicants in the Chinese context, no pre-identified concepts and theories exist, which means that new ideas could be created from data (Corbin and Strauss,2008, p. 18). Qualitative method of the grounded theory developed by Glaser and Strauss in 1967 for building theory from data was used (Glaser and Strauss, 1967). Developing a theory involves data collection, analysis, and theory building as reciprocal steps. As researchers construct the theory, they simultaneously code the information, categorize it, and relate its parts to form a logic diagram or model for testing and verification (Creswell and Brown, 1992).

Theoretical sampling is the basic principle of grounded theory (Su et al., 2020). This practice aims to collect data to maximize opportunities to develop concepts (Corbin and Strauss, 2008, p. 92). The researcher thus begins the study with a general target population, which in this case consists of the Ed.D. students enrolled in Chinese universities, and continues to sample from that group. When performing theoretical sampling, the researcher takes one step at a time, starting with data collection, followed by analysis, and followed by more data collection until a category reaches the point of “saturation” (Corbin and Strauss, 2008, p. 187).

Purposive sampling, which sets out to find people who can and are willing to provide the information (Etikan et al., 2016), was also employed to select the interviewees to provide in-depth case-oriented analysis. While selecting the interviewees, we considered certain demographic background factors, such as gender, work experience, and location, to reach informational redundancy or theoretical saturation (Sandelowski, 1995).

### Research Context

As Corbin and Strauss (2008, p. 147) stated in the research of grounded theory analysis, there is an identified population and a setting, both of that can be satisfied in a case study. Also, case studies are most suitable for answering questions that should be explored and are highly dynamic and complex (Yin, 2009). Since the study intends to document why the applicants decided to participate in the Ed.D. program, we focused on a typical provincial university in Central China as the case and selected samples from the applicants of that enrollment population. This university received permission from the Chinese Ministry of Education to recruit Ed.D. in 2018 and, in doing so, became the only university in the provincial region to conduct Ed.D program. Following the requirements established for Ed.D. candidates stated by the Chinese government, this university recruited students who had a master's degree and more than 5 years of full-time work experience in education and demonstrated considerable achievements at all levels. Applicants have to pass an entrance examination, including a written test and an in-person interview, which checks out their formal academic qualifications and previous work experience in education. In 2021, 35 applicants were permitted to enroll in the Ed.D. project. These students need to study full-time for at least 1 year, and complete their doctorate study within 4 years (or within 8 years at the most). All the students have the intention to become more experienced professionals or leaders in educational institutions.

### Instrument

The main instrument for data collection was a semi-structured interview. The first author developed the interview guide. The interview guide includes questions posed in a way that participants are free to express their experiences and perceptions. To construct our specific guide questions, we referred to several studies examining related topics of Ed.D motivation (e.g., Wellington and Sikes, 2006; Zambo et al., 2014; Wildy et al., 2015) and used best practices for survey design (Rea and Parker, 2014). An expert panel (a university professor and a senior college administrator) was invited to review the interview guide questions before the study. After being reviewed by the expert panel, the interview questions were revised based on their comments and suggestions. The modified interview guide questions are as follows: 1. What did you do before pursuing Ed.D.? 2. Why did you decide to do a doctorate? 3. Why did you choose Ed.D. instead of other doctorates? 4. What impact do you think it will have or had on your personal life? 5. What impact (if any) do you think it will have or had on your professional life? 6. What do you expect after your graduation?

The interview questions were open-ended so as not to limit the participants' ideas. These questions are designed to ask the same information in different ways to achieve triangulation, depth, and completeness. Follow-up questions were raised to enable researchers to delve into participants' thoughts. The face-to-face method allows participants to share and narrate freely and comfortably their perceptions to give rich, in-depth information, and it also helped to achieve data triangulation by using the constant comparative method, which was returning to the participants for clarification and further explication of the research topic (Snyder, 2012).

### Information Collection

From September to November 2021, 18 students pursuing Ed.D. (11 women, 7 men) at the case university were recruited to be interviewed based on the sample selection criteria mentioned earlier. They came from various professions, focused on a diverse set of research topics, and varied in terms of their geographical location and gender. Their average age was 35 years old, and they had an average of 10 years of work experience. The participants were school heads, college and university administrators, and teachers with research interests that ranged from management of the middle and primary school to higher education.

The individual face-to-face semi-structured interviews lasted for approximately 20 min each. These interviews were audio-recorded and transcribed with the interviewees' consent. The transcriptions were then coded and sorted with Nvivo12.0. The interview schedule was divided into two stages. In the first stage, 15 interviewees were selected and asked about their motives for pursuing an Ed.D. degree and what they expected to gain from their doctoral education. Their responses were preliminary analyzed to form a memorandum. Based on the theoretical sampling, another three interviews were conducted to collect more data until theoretical saturation occurred. The participants' demographic information is shown in Table 1, with the initial 15 interviewees numbered from F1 to F9 and M1 to M6 and the other three supplemental interviewees numbered as FS1, FS2, and MS1.

**Table 1:** Interviewees' demographic information.

**Interviewee**	**Sex**	**Age**	**Workplace**	**Occupation**	**Working years**	**Academic interest**
F1	Female	40	Urban Middle school	Principal	16	Leadership
F2	Female	34	Provincial University	Office Administrator	7	Human resources
F3	Female	41	Vocational College	Office Administrator	15	Entrepreneurship
F4	Female	32	Normal University	Office Administrator	7	Postgraduate education
F5	Female	38	Vocational College	Student Counselor	13	Student development
F6	Female	40	Provincial University	English Teacher	15	Professional development
F7	Female	32	Vocational College	Office Administrator	7	Instructional Supervision
F8	Female	34	Provincial University	Office Administrator	8	Instructional management
F9	Female	34	Vocational College	Teacher	8	Graduates Human Resource
M1	Male	36	Vocational College	Department Chairman	11	College transformation
M2	Male	34	Normal University	Office Administrator	8	Policies and Regulations
M3	Male	45	Vocational College	Library Director	20	College management
M4	Male	34	Provincial University	Office Administrator	7	Physical education
M5	Male	34	Provincial University	Office Administrator	7	University-enterprise cooperation
FS1	Female	35	Normal University	Office Administrator	8	Not mentioned
FS2	Female	26	Rural Primary School	Principal	5	Psychological Health Education
MS2	Male	39	Normal University	Office Administrator	14	Not mentioned

## Information Analysis

The analytical strategy employed was followed according to Corbin and Strauss' popular handbook on grounded theory. We localized and simplified the analytical strategy into three main procedures: open coding, axial coding, and integration. Nvivo software was used to facilitate the analytical procedures because of its vital coding function, enabling researchers to quickly capture the information points contained in the material source (Su et al., 2020). We first individually coded, analyzed, conceptualized, and synthesized the material of the initial 15 interviews and then analyzed three second-round interviews. No new concepts were developed during the analysis of the second-round interviews, thus we achieved conceptual saturation and could construct a coherent explanatory story (Corbin and Strauss, 2008, p. 258). To avoid the subjectivity of researchers affecting the objectivity of the coding process, the first author and the third author coded the same interview manuscript separately, and the second author checked afterward. Different opinions and disputes were regularly studied in the form of discussion and negotiation until the codes achieved refinement and agreement.

### Open Coding

Open coding refers to the practice of breaking data apart and delineating concepts to stand for blocks of raw data (Corbin and Strauss, 2008, p. 240). After considering all possible meanings, scrutinizing the context, and making constant comparisons among the first 15 interviewees' materials, we developed a broad understanding of the data, produced interpretive conceptual labels, and synthesized similar lower-level concepts as categories. Using Nvivo software to assist with the coding process and brainstorming about the data, 39 initial concepts were extracted by generalizing the primary expressions related to the motivation topic in each interview material. And then, 22 categories were constructed by taking the initial concepts with the same or similar essence together. For example, the conceptual label of “setting examples for subordinates” was generalized from F1's interview material, “I want to encourage teachers in my school to read more books by being the first to earn an Ed.D, just to show them that miracles happen,” while the conceptual label of “setting examples for children” was applied to F2's statement, “ I think a mother should set a good example for her child and let her feel that her mother is a very progressive person.” Next, the essence of the two conceptual labels was extracted and synthesized into one categorized label of “setting good examples.” Other categories were synthesized in the same way with the supervision and interaction of the three authors. Table 2 lists part of the open coding examples.

**Table 2:** Open coding examples.

**Interviewee**	**Initial account**	**Concept**	**Category**
F1	I want to encourage teachers in my school to read more books by being the first to earn an Ed.D, just to show them that miracles happen.	Setting examples for subordinates	Setting good examples
F2	I think a mother should set a good example for her child and let her feel that her mother is a very progressive person.	Setting examples for children	
F5	As a student counselor, I get stuck in a mode of following routines gradually, and I started doubting myself.	Getting stuck in work	Facing professional plight
F2	you can only feel when you are working, and you think that there are issues that need to be addressed urgently.	Facing working challenges	
F7	If you have the opportunity to do an Ed.D in university, your development prospects or what you get will be different.	Promoting personal development	Developing human capital
M5	Money and title can be taken away at any time, but your talent can never be taken away by others. I think that is one of the biggest motives to study for an Ed.D.	Improving personal intelligence	
F6	I am so tired at work. I think maybe I can change my personal situation by pursuing an Ed.D. degree, such as job adjustment or position exchange.	Getting more job options	Increasing job options
F9	In fact, my family is quite supportive, and they wish I could promote more.	Supported by family	Having interpersonal support
M3	If you study abroad, you have to stay in a foreign country for a long time. The risk of being infected with COVID-19 is relatively high, and the expenses and tuition fees are also higher.	Paying less than abroad	Appropriate cultivation mode
F3	I don't want to leave my job or resign from my workplace. I want to study and work simultaneously. This kind of Ed.D. program happens to be designed for in-service educational practitioners.	Without resigning from work	
F4	A large proportion of my classmates in postgraduate went on studying for Ph.D. after postgraduation.	Influenced by classmates	Influenced by significant others
F7	One of my colleagues passed the Doctor of Education exam. She inspired me a lot. I thought, if she could do it, why can't I try?	Influenced by colleagues	

### Axial Coding

Axial coding refers to relating concepts/categories to each other and making a more abstract hypothesis linking two categories (Corbin and Strauss, 2008, p. 259). In this coding process, we related two or more categories to a broader category, which we call the theme. We also created explanatory descriptions for these new themes. Comparative analysis and conceptual saturation were also taken into consideration; therefore, each theme was developed entirely in terms of its properties and dimensions (Corbin and Strauss, 2008, p. 255). During this coding process, the internal characteristic of the themes was explored and verified. Explanatory descriptors of themes were developed in terms of “prepositional/postpositional” dimensions and “intrinsic/extrinsic” properties. The “preposition” refers to the driving factors or conditions that currently exist, while “postposition” refers to the factors offering future benefits. “Intrinsic” property describes a person's inner desire or motivation to accomplish a goal or to complete a task (Zepeda, 2013, p. 179), while “extrinsic” property can be viewed as synergistic extrinsic motivators having a positive effect on the outcome (Fischer et al., 2019) or influenced externally from the individual, and is not necessarily for the individual's interest and enjoyment of the activity (Nota et al., 2011). Therefore, 10 themes that shed light on why students pursue an Ed.D. were developed from the 22 motivational categories on account of “prepositional/postpositional” dimensions and “intrinsic/extrinsic” properties. For example, the categories “coping with degree crisis” and “coping with external competition” indicated the current status the candidates were facing, so both of the categories have prepositional and intrinsic features; therefore, the two categories can be synthesized into a broader and more encompassing motivational theme of “resisting realistic pressure.” Table 3 presents the axial coding results.

**Table 3:** Axial coding results.

**Theme(10)**	**Category(22)**	**Dimension**	**Property**
Suiting educational background	Limited personal ability Matching front degrees	Preposition	Extrinsic
Having external support	Having organizational support Having interpersonal support Influenced by significant others	Preposition	Extrinsic
Accessible for In-service educators	Appropriate Cultivation Mode Easy Admission Tests Having Added Value	Preposition	Extrinsic
Resisting realistic pressure	Coping with degree crisis Coping with external competition	Preposition	Intrinsic
Changing current status	Facing professional plight Increasing job options	Preposition	Intrinsic
Enhancing personal capital	Developing human capital Searching social capital Enriching life experience	Postposition	Intrinsic
Pursuing academic aspiration	Realizing academic pursuits Promoting research work	Postposition	Intrinsic
Achieving career promotion	Achieving career promotion Improving working ability	Postposition	Intrinsic
Facilitating working practice	Satisfying superiors' expectations Contributing to organizations	Postposition	Extrinsic
Setting good examples	Setting good examples	Postposition	Extrinsic

### Integration

**Figure 1:**
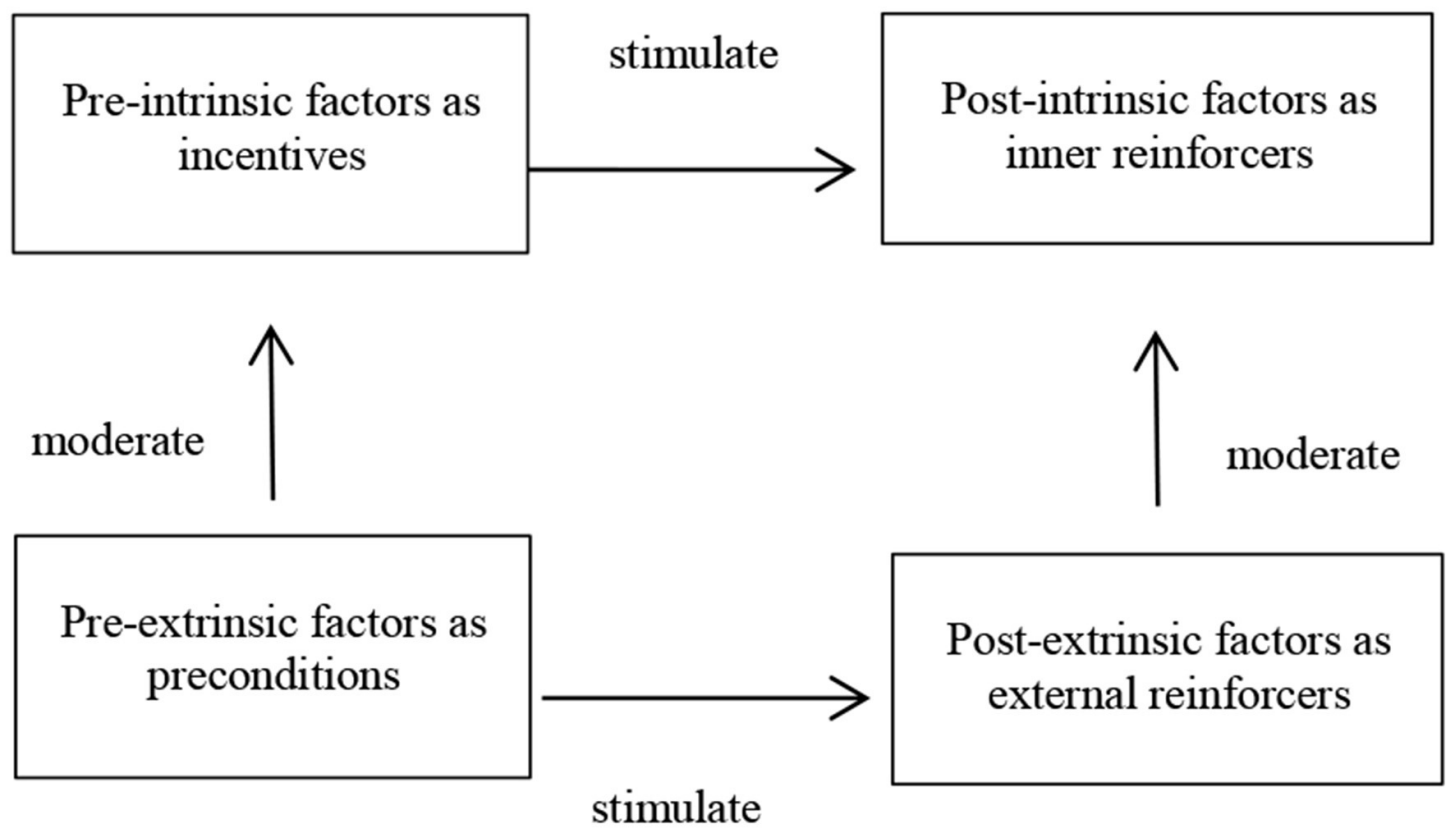
Theoretical model for the motivation factors of pursuing an Ed.D in the Chinese context.

Integration refers to linking categories around a core category and refining and trimming the resulting theoretical construction (Corbin and Strauss, 2008, p. 351). In this case, integrating themes means pulling all of the research threads together to construct a plausible explanatory framework revolving around the factors that motivate students to pursue an Ed.D in the Chinese context. According to the analysis of the axial coding process, all the motivation factors with different dimensions and properties can be grouped into four different types of motivations: pre-intrinsic, pre-extrinsic, post-intrinsic, and post-extrinsic (Table 4). Based on the interaction and logical relationship between the main categories or themes, a theoretical model for the motivation factors behind pursuing an Ed.D. in the Chinese context was constructed (Figure 1). The other three interview materials were decomposed and categorized in the same manner and no new concepts or categories emerged, which means that theoretical saturation was achieved.

**Table 4:** Types of motivations for pursuing an Ed.D. in China.

**Dimension** **Property**	**Intrinsic**	**Extrinsic**
Preposition	Resisting realistic pressure Changing current status	Accessible for in-service educators Suitable educational background Having external support
Postposition	Enhancing personal capital Pursuing academic aspiration Achieving career promotion	Facilitating working practice Setting good examples

## Results and Discussion

This research investigated the motivation factors that influence Chinese Ed.D. candidates' decision to pursue doctoral study and demonstrated four types of motivation patterns. Four motivation patterns, including pre-intrinsic, post-intrinsic, pre-extrinsic, and post-extrinsic, were presented (see Figure 1). The pre-intrinsic motives act as incentives, the post-intrinsic motives are inner reinforcers, while the pre-extrinsic motives act as preconditions and post-extrinsic motives are external reinforcers. The extrinsic motives moderate intrinsic ones, both in prepositional and post-positional dimensions. The prepositional motives stimulate post-positional ones, which leads to more diversity in the studying behaviors of Ed.D. candidates.

### Pre-intrinsic Motives as Incentives

Pre-intrinsic motivation factors signify the factors that already exist and are related to self-efficacy, which provide an essential incentive for pursuing Ed.D. In this research, two themes belong to this motivation pattern: “resisting realistic pressure” and “changing current status.” Intrinsic motivation mechanisms have been classified as being either knowledge-based or competence-based. The former is based on measures related to information acquisition, and the latter is centered on efforts related to learning skills (Baldassarre et al., 2014). For Ed.D. students, “realistic pressure” like degree crisis and fierce competition at work ask for new competence and makes students evaluate how well-suited they are for an Ed.D. program. At the same time, “current status” pushes them to acquire more learning skills and makes them determined to change.

Chinese candidates are motivated by different external pressures while deciding whether to pursue an Ed.D. Many interviewees commented that they needed to earn a doctoral degree to get promoted or cope with external competition. For instance, one interviewee said, “*In college and university, you'll find that you can't get anywhere in administration or a research field without a doctoral degree”(F4)*. Candidates from private educational institutions are facing a severer “survival crisis,” as one interviewee pointed out, “My *private college' staffing security is less than that in public colleges. It means we are more likely to lose our jobs at any time, especially when we are getting older, so we have a strong sense of crisis”(M3)*.

Some applicants also encounter difficulties in the workplace or feel bottle-necked in their career, so they hope to change their current situation by pursuing a doctoral degree. As one interviewee said*, “I am so tired at work, I think maybe I can change my personal situation by pursuing an Ed.D. degree, such as job adjustment or position exchange” (F6)*. Although these Ed.D. applicants are driven by practical reasons like improving their career status, they may face a great deal of uncertainty about the future, which leads to concerns about the gains and losses involved in the study process. That is to say, they may deviate from the academic nature of the Ed.D. degree, which is also a doctoral degree and aims to expand students' knowledge of scholarly literature to some extent (Perry, 2012a). This deviation may lead to the risk of failing to complete their doctoral studies within the scheduled time.

### Pre-extrinsic Motives as Preconditions

The pre-extrinsic motivation pattern refers to external-context factors that exist before students apply. It can mediate between situational factors and boost Ed.D. candidates' confidence to apply. In this study, external-context factors include whether a program is accessible for in-service educators, whether the candidates' educational background is suitable for the Ed.D. program, and whether an applicant has external support.

Ed.D. programs primarily target in-service education practitioners with more than 5 years of working experience. As Wildy et al. (2015) explained, these students are “likely to be mature-aged, mid-career professionals who are keen to progress in their workplace” and can be “characterized as time-poor and experience-rich.” Regarding the doctorate entrance examination in China, candidates for Ed.D. do not need to score as high as those for Ph.D. In addition, the entrance exam content mainly focuses on pedagogy and related disciplines, which is beneficial for applicants who have been working in the educational field for a long time. Many Ed.D. applicants assessed their educational background and decided to pursue an Ed.D. rather than a Ph.D.: “*This kind of professional degree is more suitable for those who have been working for so many years. If you apply for an academic doctoral degree, it may be a little more difficult” (M1)*.

In China, the Ed.D. program was designed by the government in a way that catered to the in-service educational practitioners who wanted to pursue a doctorate. The candidates who were pursuing their Ed.D. were appreciative of the economical characteristics of the policy: “*[The Chinese Ed.D. program] is more economical than studying abroad. If you study abroad, you have to stay in a foreign country for such a long time. The risk of being infected with COVID-19 is relatively high, and the expenses and tuition fees are also higher”(M3)*.

Because Chinese Ed.D. programs are designed for in-service people, most of the students have to condense their working time to study. In the face of such time and role pressure, external support is vital for Ed.D students. Some interviewees said they receive support from their leaders, “*I think my leader cares for me and give me the freedom to deal with my work and study“ (F8)*. Others commented that they receive support from their families, “*I can't do my Ed.D. without the support of family members, especially my mother-in-law, who helps me take good care of my five-year-old daughter” (F9)*. Some respondents mentioned that they had a “significant person” in their lives, who exerted a subtle influence on them, “*My brother supports my decision to pursue a doctoral degree because he is a professor and a Ph.D. himself, and he will give me some guidance and help in my study”(F3)*.

As these responses show, Ed.D. candidates function as workers and students, parents and spouses, and professionals and practitioners under different social contexts. So they are experiencing role conflict, which means that they are confronted by situations in which they may be required to play two or more roles that are in direct opposition to each other (Van Sell et al., 1981). Apart from the role conflict, Ed.D. candidates must grapple with higher requirements in their work environments and academic platforms, especially when it comes to acquiring professional titles and better salaries. Thus, they may face intense stress and imbalance and try to moderate their different roles, which may interfere with their determination to complete the Ed.D. degree.

### Post-intrinsic Motives as Inner Reinforcers

The post-intrinsic motivation pattern indicates the factors that benefit the future self, reinforcing the candidates' beliefs of promoting personal life through pursuing Ed.D. In this study, this pattern of motivation consists of three themes: enhancing personal capital, achieving career promotion, and pursuing academic aspirations.

Although Chinese Ed.D. applicants have already entered the job market, they still expect to receive higher returns through the improvement of personal capital. Social capital has a substantial independent influence on income, net of human capital, and position level (Boxman et al., 1991). Moreover, social capital, like peer support groups and connections, facilitates outcomes, such as professional advancement, information acquisition, and identity development (Sweitzer, 2009). Ed.D. candidates want to enhance their social capital and improve human capital through Ed.D. study. As one interviewee explained, “*Because the Ed.D. program allows us to acquaint with many students who are excellent in education management at all levels and in various types of schools, I think it is a rare opportunity”* (M1). Another interviewee said, “*Money and title can change or be taken away at any time, but your talent can never be taken away by others. Therefore, it is one of the biggest motivation for me to improve my skills through studying for an Ed.D”* (M5). The accumulation of human and social capital can eventually help Ed.D. students improve their position at work. One interviewee emphasized this point when they said, “*In terms of getting a professional title and career promotion, as long as you get this Ed.D. degree, you can definitely break through this ceiling”* (F4).

Many Ed.D. candidates also experience a dichotomy between theory and practice. Luckily, theory and practice are rigorously connected in Ed.D. programs, and this praxis is both research-based and research-driven, which underpins the improvement of the learners' professional practice (Taysum, 2007). Some students thus hope to strengthen their research abilities and pursue their academic aspirations during their doctoral studies. As one interviewee expressed, “*I have the dream of pursuing doctoral study. On the one hand, I want to pursue a doctoral degree. On the other hand, I want to achieve my academic aspirations”*(M2). As these examples show, Ed.D. students are not only driven by realistic factors, such as “earning more” or “being promoted at a quicker pace,” but also influenced by ideological factors, such as academic dreams, interests, and values.

### Post-extrinsic Motives as External Reinforcers

Post-extrinsic factors may result in favorable external outcomes. In this study, the themes “facilitating working practice” and “setting good examples” come into play in this respect. The Ed.D. programs try to meet the needs of practitioners who work in various contexts while preparing students to apply theory to their practice and learn how to solve better on-the-ground problems (Zambo et al., 2014). The Ed.D. programs are thus closely linked with working practice, which is why some educational practitioners decide to pursue Ed.D. As one interviewee said, “*I hope I can learn more and make greater contributions to my work”* (F8). Another participant echoed this idea when they said, “*I don't know how to deal with many problems at work, so I hope to come here to learn”*(F5).

As Hofstede (2011) mentioned, China is a country that embraces a collectivist culture, in which people from birth are integrated into strong, cohesive in-groups. An emphasis is placed on the interdependent relationship between people and the collective, and people acquire their self-identity through group membership. This is especially the case with educational practitioners because they are employed by the government and more ingrained in the collective complex, which leads them to boost their in-groups and organizations by improving themselves. As one interviewee said, “*My college develops quite slowly, and especially lacks high-level talented people. Honestly, an important reason I pursue my Ed.D. is to look outside and see how others develop”*(M1).

Many Ed.D. applicants are women. Although it is more difficult for women to balance study, family, and work than men, Chinese women witnessed a great change in recent years. Under the guidance of academic examples, women attempt to pursue educational equality to achieve self-improvement. They not only gain strength from role models but also aspire to become role models themselves. As one interviewee said, “*I think a mother should set a good example for her child and make her feel that her mother is a very progressive person”* (F2). Another interviewee discussed this point when they mentioned, “*I (as a principal) want to encourage teachers in my school to read more books by being the first to earn an Ed.D., just to show them that miracles happen”* (F1).

### Extrinsic Motives Moderate Intrinsic Ones

Ed.D. candidates' extrinsic motivators can have synergistic effects on their intrinsic motivators. Self-determination theory (SDT) may help explain how the socio-context variables nurture Ed.D. candidates' intrinsic motivation. SDT identifies three types of self-perception (autonomy, competence, and relatedness) that affect the motivation of students (Mason, 2012). When people have autonomy, their behavior is self-determined, and they have the option of choosing what they do. When intrinsic motivation was attained, people would become more self-determined (Deci and Ryan, 2013). Research has shown that graduate students' autonomy is related to their satisfaction with graduate programs and degree completion (Mason, 2012). Most Ed.D. candidates are satisfied with the program because it is tailored to the educational practitioners in the Chinese context. This external component has been transformed into Chinese candidates' internal self-regulated learning needs, as one interviewee said, “*I don't want to leave my job or resign from my workplace. I want to study and work simultaneously. This kind of Ed.D. program happens to be designed for in-service educational practitioners”* (F3).

Competence is understanding how to achieve desired outcomes and having the self-efficacy to carry out the actions required in a specific context (Deci et al., 1991). Ed.D candidates, who have more excellent expertise in educational practice, are optimistic about completing the Ed.D. program, benefiting from more extensive networks in the field, and becoming better equipped to address a wide range of issues in their workplaces. Thus, their competence in doing a good job urges them to be a better person. For instance, one interviewee made a wish: “*I am looking forward to solving practical problems in my work through my doctoral study and research in education”* (F2).

A feeling of relatedness taps into a fundamental need to be connected, accepted, and valued by others (De Clercq et al., 2021). Mason (2012) explained that relatedness to family, peers, teachers, and advisors have an individual effect on motivation. Support from significant others thus transformed into an intrinsic pattern to give Ed.D. candidates the courage and impetus they need to pursue the program. An interviewee expressed her gratitude to her teacher: “*My former supervisor, Doctor Yi, sent me a message asking me to pay attention to the recruitment information on the postgraduate website and encouraged me to take the exam. He thought I was qualified for the program”* (F6).

### Prepositional Motives Stimulate Postpositional Ones

Some of the prepositional motivators are linked to feelings of pressure and dissatisfaction with work positions that lead to the postpositional motivators of wanting to change or improve something in the future. This hypothesis comes from the features of the Ed.D. program. It enables students to kill at least two birds with one stone because the assignments and research can be oriented toward work-related issues and concerns (Wellington and Sikes, 2006). Some Ed.D. candidates thus want to change their current working status by progressing in their workplace while facilitating their professions and academics. One interviewee said, “*Although my work has encountered a bottleneck and I moved to teaching position, I don't exclude doing administrative work after I get a doctorate. I am still willing to return to the administrative position, and I will do it easier and better”* (M6).

Another example is linked to the themes of “resisting realistic pressure” and “enhancing personal capital.” Improving human capital and social capital can help confront realistic pressure, such as peer competition and social involution. Social capital that takes the form of peer support groups and academic networks is critical to ensuring success and support beyond an academic advisor or supervisor (Leonard and Becker, 2009). Sources of human capital, such as academic skills and practical knowledge, are significant in improving professional practice. As an interviewee explained, “*I am not outgoing, but here I greatly improved my interpersonal communication skills. At the same time, I felt I had absorbed a lot of knowledge that I did not know before. It gives me a great deal of confidence and resources”* (F6).

## Conclusion

This study used qualitative research to explore Chinese Ed.D. candidates' motivation for pursuing doctoral education. For most people, the decision to earn an Ed.D. was motivated and informed by various factors that shed light on their various identifies and needs. After analyzing the interview materials using grounded theory, four motivation patterns emerged: pre-intrinsic, pre-extrinsic, post-intrinsic, and post-extrinsic. The four motivation patterns were organized to construct a theoretical model with a mutual logical relationship. In sum, the research findings are significant because they shed new light on motivations for pursuing an Ed.D. in China from the students' perspective. This study also provides some suggestions for Ed.D.-related groups.

Ed.D. candidates need to recognize the diverse motivational factors that influence their choices and carry out effective transformation when appropriate. The motivation factors of the Ed.D. students are shaped by individual educational backgrounds and working experiences, reflecting significant differences among individuals. Some Ed.D. candidates are motivated by solid utilitarian and realistic factors that can interfere with learning and academics. The completion ratio of the Ed.D. students is lower than that of Ph.D. students in China. The Ed.D. students should thus transform extrinsic motivation factors into intrinsic motivation factors because an intrinsic motivation for learning progress allows a student to tackle more difficult learning problems in progressive ways (Triesch, 2013).

Universities and supervisors should better understand the learning demands of the Ed.D. students and work to improve their learning satisfaction. Up to this point, the voices of Ed.D. students have not been reflected in research about their needs and demands, and they have received little support in their unique learning environments (Jung, 2018). For example, for those Ed.D students primarily motivated by the realization of academic ideals, tutors should focus on strengthening their research abilities and academic skills. For those who view improving their workability as their main motivation factor, universities and tutors should fully respect their willingness to learn independently and provide more opportunities for practical learning and case discussion. More importantly, supervisors should offer personal and professional support to Ed.D. students and look after their emotional wellbeing (Kumar and Kaur, 2019).

Chinese educational governors should strengthen investigating Ed.D. programs and optimize the training mechanism. The Ed.D. students who have full-time employment struggle to balance work and study, especially female Ed.D. students, who experience a tension between their roles as wife/mother and student (Brown and Watson, 2010). When encouraging the expansion of Ed.D. in China, the government should also consider the learning difficulties, graduation pressures, work constraints, and family troubles that Ed.D. students may bear, and explore feasible ways to solve those problems from the perspective of humanistic care.

Chinese educational researchers should pay more attention to the generation mechanism behind the motivation factors of Ed.D. students and the specific patterns of motivation that exist among Ed.D. students. For example, researchers need to be more aware of the “dual lives” that many female Ed.D. students lead and recognize that their motivation and learning behavior deserve more profound attention. In addition, as a country that is working to cultivate Ed.D. students on a massive scale, China needs to learn from the experiences of other countries and develop a stronger voice in this field of research.

This study has several limitations, and future studies are needed to address these issues. First, the study only focused on one university case. Future studies need to include more universities with different enrollment demands and learning environments. Second, though the sample interviewees had diverse educational backgrounds and work experiences, the changing ways of their motivations throughout the program are ignored. Future studies need to conduct more interviews over time to determine whether students change their perceptions of their motivations for earning Ed.D. degrees. Third, this research considers various motivation factors but did not discuss which factor exerts the most influence on the candidates. Future studies need to explore how the motivational mechanism works and affects Chinese students learning process.

## Data Availability Statement

The original contributions presented in the study are included in the article/supplementary material, further inquiries can be directed to the corresponding author.

## Ethics Statement

The studies involving human participants were reviewed and approved by School of Educational Science, Hunan Normal University. Written informed consent for participation was not required for this study in accordance with the national legislation and the institutional requirements.

## Author Contributions

WG performed the data analyses and wrote the manuscript. WW helped perform the analysis with constructive discussions. CX contributed to the methods implementation and manuscript preparation. All authors contributed to the article and approved the submitted version.

## Funding

This work was supported by the Education Department of Hunan Province, China, Hunan Province' Academic Degree and Postgraduate Teaching Reform Research Project, the construction of quality evaluation model and guarantee mechanism of Ed.D. students in China, No. 2021JGYB223.

## Conflict of Interest

The authors declare that the research was conducted in the absence of any commercial or financial relationships that could be construed as a potential conflict of interest.

## Publisher's Note

All claims expressed in this article are solely those of the authors and do not necessarily represent those of their affiliated organizations, or those of the publisher, the editors and the reviewers. Any product that may be evaluated in this article, or claim that may be made by its manufacturer, is not guaranteed or endorsed by the publisher.
